# Novel model of pyroptosis-related molecular signatures for prognosis prediction of clear cell renal cell carcinoma patients

**DOI:** 10.7150/ijms.88301

**Published:** 2024-01-01

**Authors:** Jiaxin Chen, Runyi Jiang, Wenbin Guan, Qifeng Cao, Yijun Tian, Keqin Dong, Xiuwu Pan, Xingang Cui

**Affiliations:** 1 Department of Urology, Xinhua Hospital, Shanghai Jiaotong University, School of Medicine, 1665 Kongjiang Road, Shanghai 200092, China.; 2 Department of Urology, Third Affiliated Hospital of the Second Military Medical University, Shanghai 200433, China.; 3 Spinal Tumor Center, Department of Orthopaedic Oncology, Changzheng Hospital, Second Military Medical University, Shanghai, 200003, China.; 4 Department of Pathology, Xinhua Hospital, Shanghai Jiaotong University, School of Medicine, Shanghai 200092, China.

**Keywords:** pyroptosis, clear cell renal cell carcinoma, molecular signatures, prediction model

## Abstract

**Background:** Pyroptosis is a programmed death mode of inflammatory cells, which is closely related to tumor progression and tumor immunity. Clear cell renal cell carcinoma (ccRCC) is the major pathological type of renal cell carcinoma (RCC) with poor prognosis. Many theories have tried to clarify the mechanism in the development of ccRCC, but the role of pyroptosis in ccRCC has not been well described. The main purpose of this study is to explore the role of pyroptosis in ccRCC and establish a novel prognosis prediction model of pyroptosis-related molecular signatures for ccRCC.

**Methods:** In the present study, we made a systematical analysis of the association between ccRCC RNA transcriptome sequencing data from The Cancer Genome Atlas (TCGA) database [which included 529 ccRCC patients who were randomized in a training cohort (n=265) and an internal validation cohort (n=264)] and 40 pyroptosis-related genes (PRGs), from which four genes (CASP9, GSDME, IL1B and TIRAP) were selected to construct a molecular prediction model of PRGs for ccRCC. In addition, a cohort of 114 ccRCC patients from Shanghai Eastern Hepatobiliary Surgery Hospital (EHSH) was used as external data to verify the effectiveness of the model by immunohistochemistry. Moreover, the biological functions of the four PRGs were also verified in ccRCC 786-O and 769-P cells by Western blot (WB), CCK-8 cell proliferation, and Transwell invasion assays.

**Results:** The model was able to differentiate high-risk patients from low-risk patients, and this differentiation was consistent with their clinical survival outcomes. In addition, the four PRGs also affected the ability of cell proliferation and invasion in ccRCC.

**Conclusion:** The prediction model of pyroptosis-related molecular markers developed in this study may prove to be a novel understanding for ccRCC.

## Introduction

Renal cell carcinoma (RCC) is a common malignant tumor of the urinary system originating from the parenchymal epithelium of renal tubules 1. It is estimated that RCC caused about 431,288 new cases and 179,368 deaths worldwide in 2020 2, seriously endangering human health. Most RCC patients were found to be in an advanced stage at diagnosis, which is the main reason for the poor prognosis of the disease 3. Clear-cell RCC (ccRCC) is the most common subtype, accounting for about 70% of all RCC cases 4. It is considered an immunogenic tumor by inducing immune dysfunction and eliciting infiltration of immune-inhibitory cells such as regulatory T (Treg) cells and myeloid-derived suppressor cells (MDSCs) into the tumor microenvironment (TME) 5. Given the clinically observed heterogeneity of ccRCC 6-8, many studies have revealed the intra-tumor and inter-tumor heterogeneity of ccRCC through tumor genomics 9-12. The complexity of the mechanism has always been a challenge in biological research.

Pyroptosis, a form of proinflammatory cell death, has been identified as caspase-1-mediated monocyte death and is accompanied by interleukin-1β (IL1B) and interleukin-18 (IL-18) secretion 13. Pyroptosis is a regulated cell death that relies on gsdermin family proteins to form membrane pores in the plasma membrane 14. Cleavage of gasdermin D (GSDMD) by caspase-1, -4, -5, or -11 defines the canonical pyroptosis pathway 15-17. Caspase-3 was reported to specifically cleave gasdermin E (GSDME), leading to chemotherapy drug-induced normal-tissue damage or viral infection-mediated secondary necrosis 18,19, revealing that GSDME also has pyroptotic potential.

Interestingly, emerging evidence indicated that pyroptosis is related to tumor immunity 20,21. Many pyroptosis-related genes are highly expressed in gastrointestinal tumors. For example, gasdermin B (GSDMB) was reported to be highly expressed in digestive system epithelial cell-derived tumor cells, and the induction of pyroptosis by GSDMB could enhance antitumor immunity 22, suggesting a potential strategy for anticancer therapy by inducing pyroptosis in tumor cells. However, few reports have explicated the effect of pyroptosis in ccRCC. In the present study, we constructed a prediction model of pyroptosis-related molecular markers. Moreover, several *in vitro* experiments (contain WB, CCK-8 cell proliferation, and Transwell invasion assays) were conducted to reveal that the four PRGs (CASP9, GSDME, IL1B and TIRAP) selected in our study may affect the ability of cell proliferation and invasion in ccRCC.

## Materials and Methods

### Data download, processing, and screening

A total of 529 CCRCC patients with mRNA sequencing data and clinical information were obtained from The Cancer Genome Atlas (TCGA) database. They were randomized into two cohorts: a training cohort (n=265/50%) and an internal validation cohort (n=264/50%), and all data of transcriptional sequencing were analyzed as fragments per kilobase per million (FPKM). In addition, pyroptosis-related genes (PRGs) were collected from previous studies (Table 1). The clinical data of training cohort and internal validation cohort can been respectively seen in Supporting information 1 and Supporting information 2.

### Tumor classification based on PRGs

Univariate Cox regression analysis was used to explore the association between prognosis and expression of 40 PRGs in ccRCC patients of the training cohort, and PRGs with p < 0.05 were subjected into tumor classification by the non-negative matrix factorization (NMF) method with R package “NMF”. The optimal k value used to determine the number of subclusters was determined by the cophenetic.

### Construction and evaluation of a prognostic signature based on PRGs in the TCGA ccRCC training cohort

PRGs related to the prognosis of ccRCC patients were also sent into the analysis of least absolute shrinkage and selection operator (LASSO) regression before undergoing multivariate Cox regression analysis. The above two steps were implemented by R package “glmnet” and “survival”, respectively. After that, each PRG obtained a β value for constructing a risk score formula. The formula for each patient is as follows:



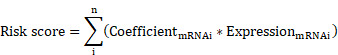



Patients in the training cohort were classified into a low-risk group and a high-risk group based on the median risk score. Furthermore, the areas under the receiver-operator characteristic curves (ROC) were utilized to assess the accuracy of the prognostic signature by using R package “survivalROC”. Patients were reordered based on the risk scores to plot the risk curve, survival status-related scatterplot and Kaplan-Meier (KM) curves, which simply identified different survival statuses for the two risk groups. Meanwhile, a heatmap demonstrating the differently expressed PRGs in the low- and high-risk groups was plotted.

### Independent prognostic analysis of the risk scores and functional analysis between the two risk groups

Combined with other clinical factors, the risk scores were further verified by prognosis analysis. After univariate and multivariate Cox regression analyses, significant clinical factors (p<0.05 in both analyses) were selected to build a nomogram (R package “survivalROC”) to predict survival probability for ccRCC patients. In addition, AUC and calibration curves were used to evaluate the accuracy of the nomogram. Differently expressed genes (DEGs) in low- and high-risk groups were determined by “limma” R package according to the criteria (|log2FC| ≥ 1 and adjusted p-value < 0.05), which is the basis of Gene Ontology (GO) enrichment analysis by applying “ClusterProfiler” package. In addition, the activity of 13 immune-related functions in high-risk group and low-risk group was calculated with single-sample gene set enrichment analysis (ssGSEA) in the "GSVA" R package.

### Validation of the developed signature in an internal cohort

Similar to the training cohort, each patient in the validation cohort got a risk score using the same formula, which helped divide the 264 patients into a low-risk group and a high-risk group according to the median risk score. Additionally, the risk curve, survival status-related scatterplot and KM curves were also used to validate the distinct prognoses in the two risk groups, and the AUC was used to evaluate the predictive ability of the gene signature. Univariate and multivariate Cox regression helped identify whether the risk scores were independent clinical risk factors in the validation cohort. Ultimately, GO enrichment and immune-related functions were analyzed to explore the difference in biological function between the two groups.

### Patients and tissue samples from the Eastern Hepatobiliary Surgery Hospital (EHSH)

114 ccRCC patients received partial or radical nephrectomy between January 2015 and February 2016 at the Department of Urology of EHSH affiliated to the Second Military Medical University (Shanghai, China). These ccRCC tissues and related paired adjacent tissues were collected from the 114 EHSH ccRCC patients, and then made into tumor microarrays. The clinical data of EHSH cohort can been seen in Supporting information 3.

### Immunohistochemistry (IHC) assay

The 114 EHSH ccRCC tissue chip was firstly added dropwise with diluted antibodies of CASP9 (ab32539, 1:1000, Abcam, USA), GSDME (13075-1-AP, 1:1000, Proteintech, USA), IL1B (ab283818, 1:1000, Abcam, USA) and TIRAP (NBP2-95138, 1:1000, Bio-Techne, USA). Then the 114 EHSH ccRCC tissue was add anti rabbit HRP, horseradish enzyme labeled streptavidin working solution and DAB successively, and finally observe under the optical microscope. And the staining intensity were scored as 0 (negative), 1 (weakly positive), 2 (moderately positive), and 3 (strongly positive). Calculation of the final evaluation criteria for positive cell frequency are as follows: staining intensity score (0-3) × staining area (0-100), full score: 300 points. The detailed information on IHC score can be seen in Supporting information 3.

### RNA interference and overexpression plasmid

The RNA oligo sequences of the genes in the model are as follows:

CASP9, SS: AGAGGUUCUCAGACCGGAAAC, AS: UUCCGGUCUGAGAACCUCUGG;

GSDME, SS: GAAUGACUCUGAUAAGUUACA, AS: UAACUUAUCAGAGUCAUUCAG;

IL1B, SS: GCGUGUUGAAAGAUGAUAAGC, AS: UUAUCAUCUUUCAACACGCAG.

The coding sequence of TIRAP was inserted into pcDNA3.1(+) by using the QuickFusion cloning kit (Biotool, USA).

### Cell culture

ccRCC 786-O and 769-P cells were obtained from the American Type Culture Collection (ATCC, Manassas, VA, USA) in 2016. 786-O and 769-P cells were routinely maintained in RPMI-1640 (added with 1% penicillin-streptomycin and 10% fetal bovine serum) (Gibco, USA) in a humid atmosphere with 5% CO_2_ at 37℃. The siRNA of CASP9, GSDME and IL1B were transfected with Lipofectamine RNAiMAX Reagent (Thermo Fisher Scientific, USA). The overexpression plasmid of TIRAP was transfected with Lipofectamine 2000 Reagent (Thermo Fisher Scientific, USA).

### Western blot (WB) analysis of the model

The total protein in cells (ccRCC 786-O and 769-P) was extracted by SDS-polyacrylamide gel electrophoresis (SDS-PAGE) and then transferred to polyvinylidene fluoride (PVDF) membrane (Thermo, USA). After that, the PVDF membrane was incubated with the diluted antibodies: CASP9 (ab32539, 1:1000, Abcam, USA), GSDME (13075-1-AP, 1:1000, Proteintech, USA), IL1B (ab283818, 1:1000, Abcam, USA), and TIRAP (NBP2-95138, 1:1000, Bio-Techne, USA). Then the PVDF membrane was washed and incubated with the diluted horseradish peroxidase-conjugated goat anti-rabbit (1:2000, Santa Cruz, USA). The b-actin (ab8226, Abcam, USA) was used as the loading control.

### CCK-8 cell proliferation assay

After digestion, counting and centrifugation, 786-O and 769-P cells (containing the wild type (WT) control group and siRNA knockdown or overexpression group) were seeded into the 96-well plates and cultured for 24h (3000 cells per well). Then the Cell Counting Kit 8 (Biotool, USA) was added into these cells (10μL CCK-8 reagent per well) and cultured for 2h, then measured the absorbance at 450 nm per well.

### Transwell cell invasion assay

Firstly, the precooled matrix gel was diluted with serum free medium in a ratio of 1:8, and then added to the bottom of the transwell chamber (50μL matrix gel per well). Afterwards, cells were inoculated onto the transwell chamber and the total number of cells per well was 4 × 10^4^ / mL. RPMI-1640 (added with 15%FBS) was added into the bottom of the chamber. After 48-h tranquillization at 37°C and 5% CO_2_, cells were fixed in methanol solution and stained with crystal violet successively, and then washed the transwell chamber with tap water. Finally, the number of membrane-passing cells was counted under the light microscope (five randomly selected fields).

## Results

### Preliminary screening of key genes for pyroptosis

**Figure 1:**
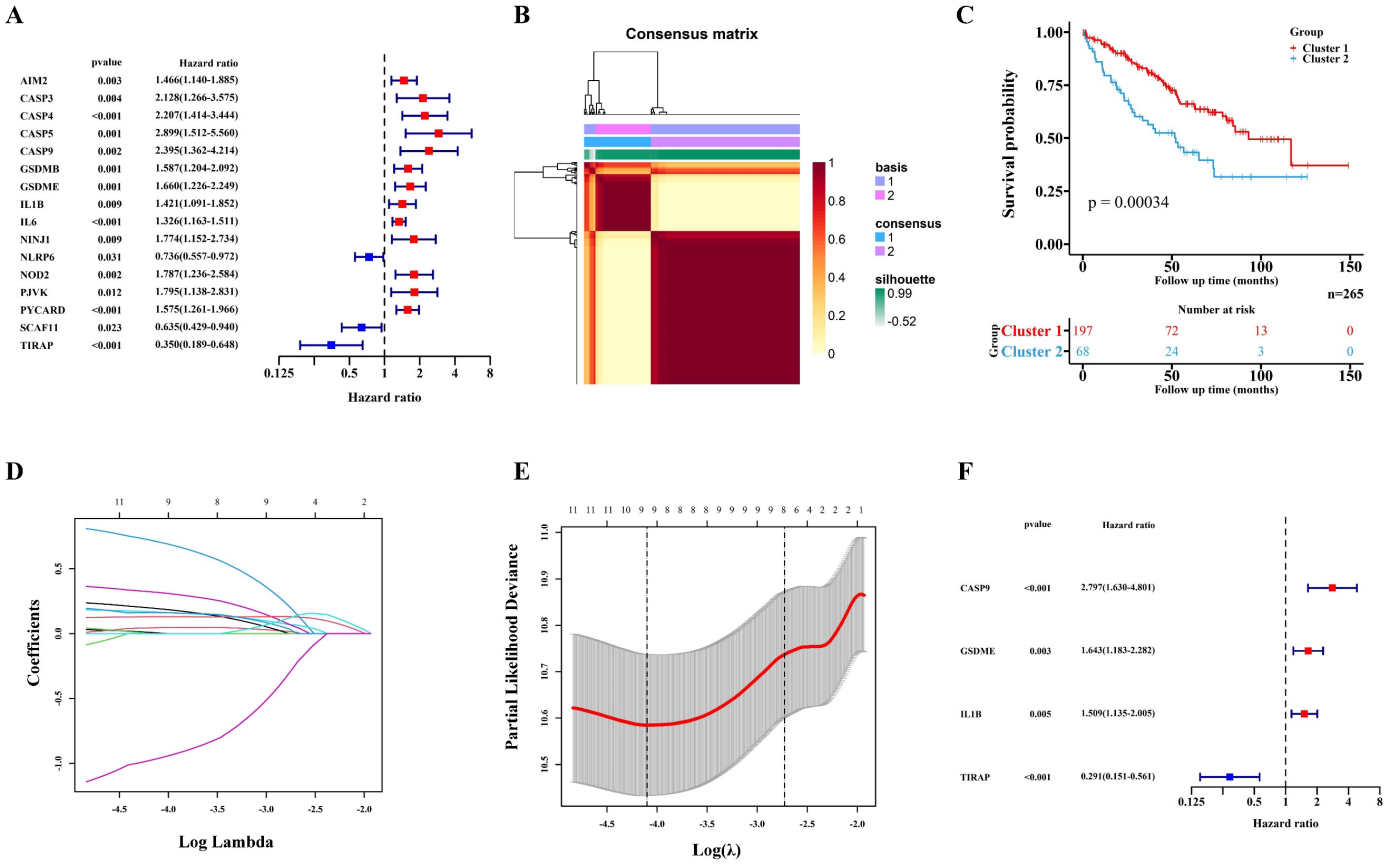
Construction of the prognosis prediction model depends PRGs in TCGA ccRCC training cohort. (A) 16 PRGs related to the clinical prognosis of ccRCC were screened from 40 PRGs by univariate cox regression. (B) Two subgroups were identified by the NMF. (C) The Kaplan-Meier survival analysis of the related patients in two different subgroups. (D,E) 16 preliminary candidate PRGs under the LASSO analysis. (F) 4 final PRGs of the model (CASP9, GSDME, IL1B and TIRAP) screened out by multivariate cox regression.

529 ccRCC transcriptome data of TCGA were randomly divided into two groups: a training cohort (n = 265) and a validation cohort (n=264). The training cohort data were analyzed and modeled with 40 PRGs. Firstly, 16 pyroptosis key genes related to the clinical prognosis of the training cohort were screened, including 13 promoting genes and 3 protective genes (Figure 1A). After that, the preliminary model was divided into two different groups (a high-grade group and a low-grade group) by the NMF algorithm (Figure 1B; Figure S1A-B). There were significant differences in survival and prognosis between the two groups (Figure 1C).

### Selection of key PRGs and establishment of the prediction model

**Figure 2:**
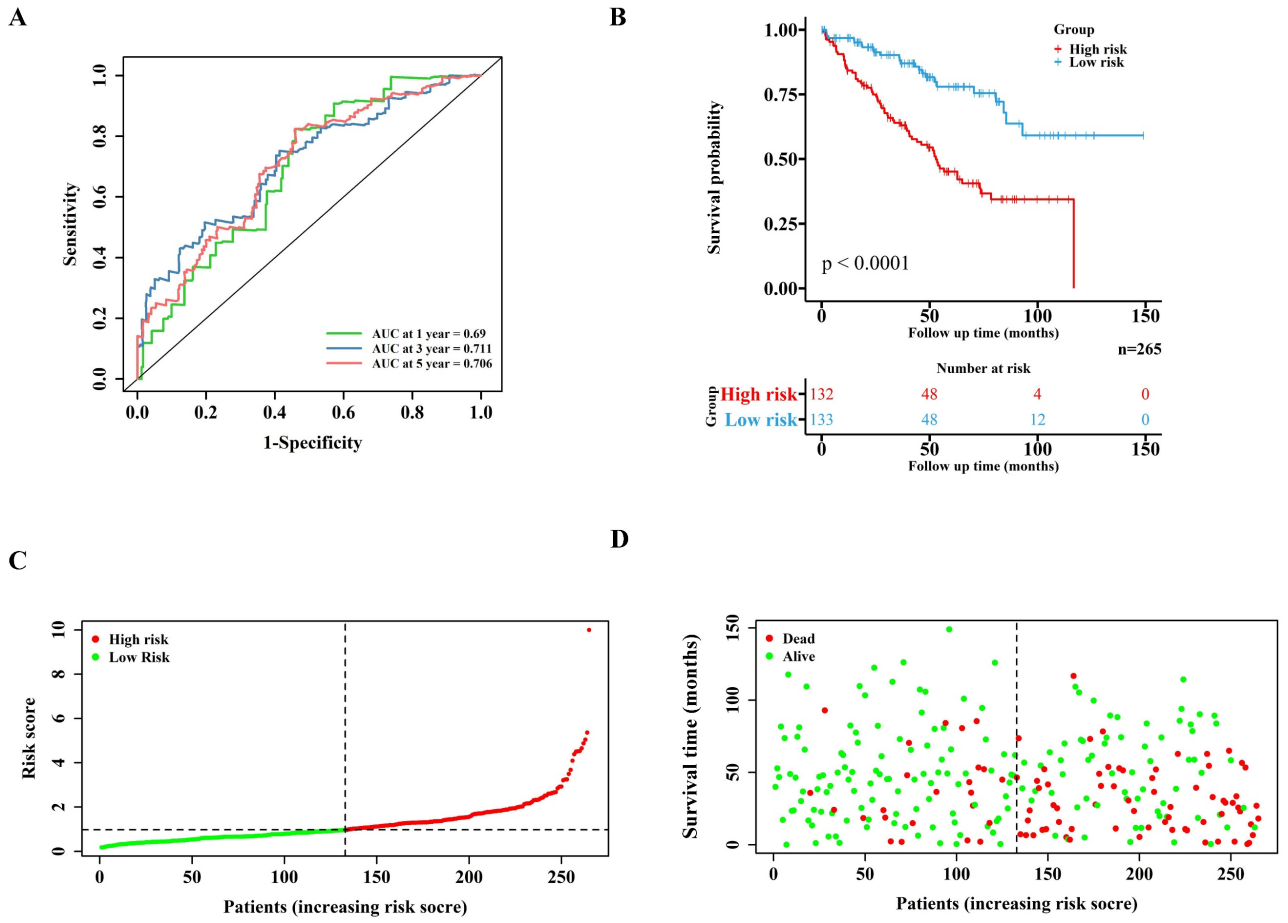
Validation of the PRGs prediction model in training cohort. (A) Time-dependent ROC curve (1 year-, 3year-, 5year-) of the PRGs prediction model in training cohort. (B) The Kaplan-Meier survival analysis of the training cohort patients in high-risk and low-risk groups. (C,D) Scatter diagram visualizing the distribution of the risk score and survival time of training cohort patients in the prediction model.

Lasso regression (Figure 1D-E) and multivariate cox regression (Figure 1F) were used to finally select four key molecules related to pyroptosis: CASP9, GSDME, IL1B and TIRAP. The survival prognosis of the four genes for the training cohort was also shown by KM curves Figure S2A). Using these four key molecules, the risk prediction model of PRGs in ccRCC was established. The sensitivity of the model is shown in in Figure 2A. The formula of our model as follow: Risk score = a × (1.028665075621620) + b × (0.496439639415188) + c × (0.411374794253998) + d × (-1.236071649587060) (a,b,c,and d represent the mRNA expression of CASP9, GSDME, IL1B and TIRAP respectively). The risk score of the model was verified in the training cohort. The KM curves, scatter diagram and heatmap all showed that patients with high-risk scores had poorer survival prognosis, and those with low-risk scores had better prognosis (Figure 2B-D; Figure S2B).

### Correlation between the model and clinical indicators and the immune-related pathway score of the model

**Figure 3:**
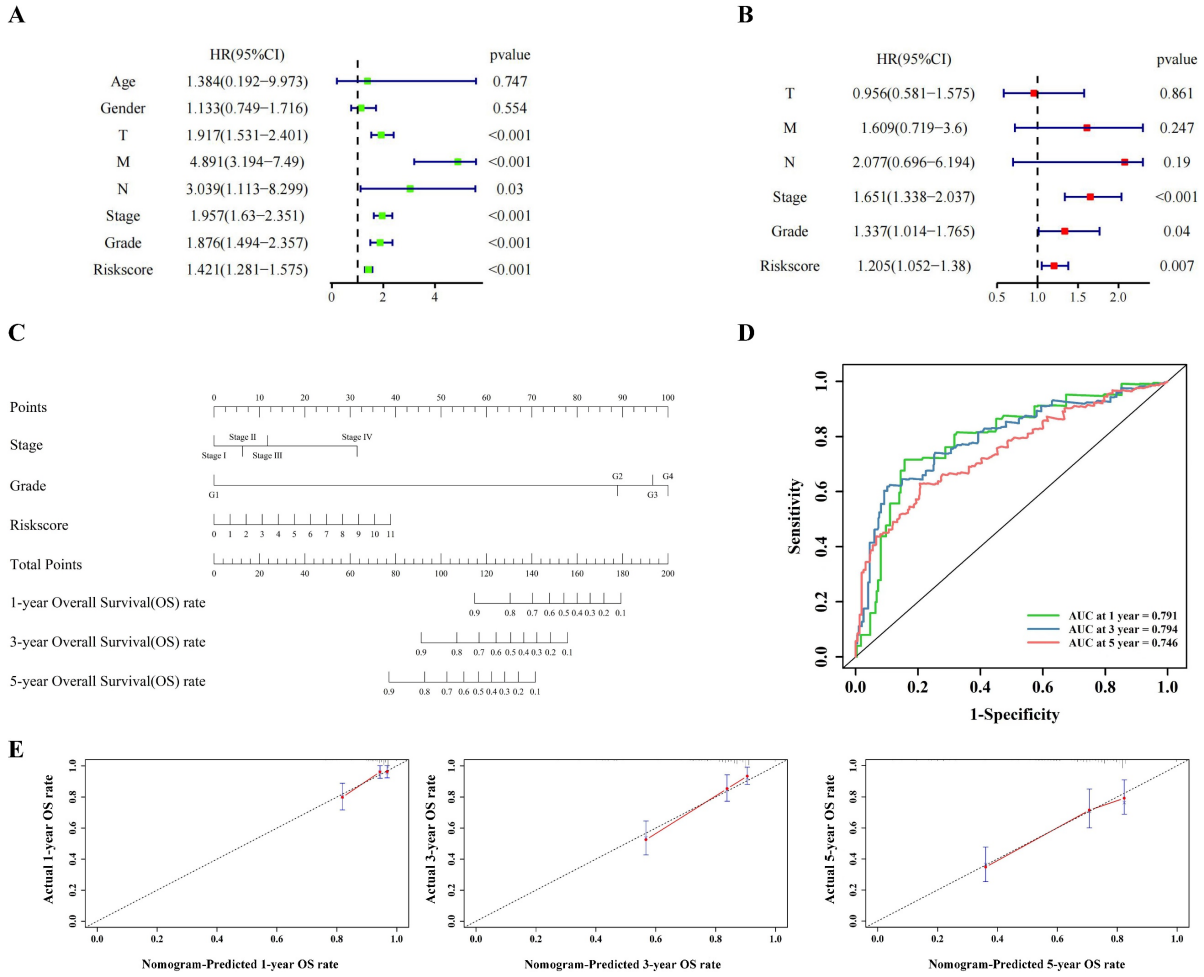
The clinical indicators analysis of the model in the training cohort. (A,B) Independent clinical indicators screened out by univariate cox regression and multivariate cox regression. (C) Nomogram showing the correlation between the risk score and clinical indicators. (D) Time-dependent ROC curve (1 year-, 3year-, 5year-) of the nomogram model in training cohort. (E) Calibration of the nomogram at 1 year-, 3year-, 5year- OS rate in the training cohort.

Univariate cox regression (Figure 3A) and multivariate cox regression (Figure 3B) were used to analyze the correlation between the model and the clinical indicators. As shown by the nomogram, the model was significantly correlated with tumor stage, tumor grade and riskscore (Figure 3C). The ROC curve also demonstrated that the nomogram prediction model was sensitive (Figure 3D). The 1-, 3- and 5-year survival rates predicted by nomogram are shown in the line chart (Figure 3E). Gene Ontology (GO) enrichment analysis (Figure S3A) and Kyoto Encyclopedia of Genes and Genomes (KEGG) analysis (Figure S3B) showed that the model was enriched in immune-related pathways, and ssGSEA (Figure S5A) showed that the scores of immune related pathways in high-risk group were higher than those in low-risk group. These results may suggest that pyroptosis is closely related to immune function in ccRCC.

### The risk score of the centralized validation model in the internal validation cohort

**Figure 4:**
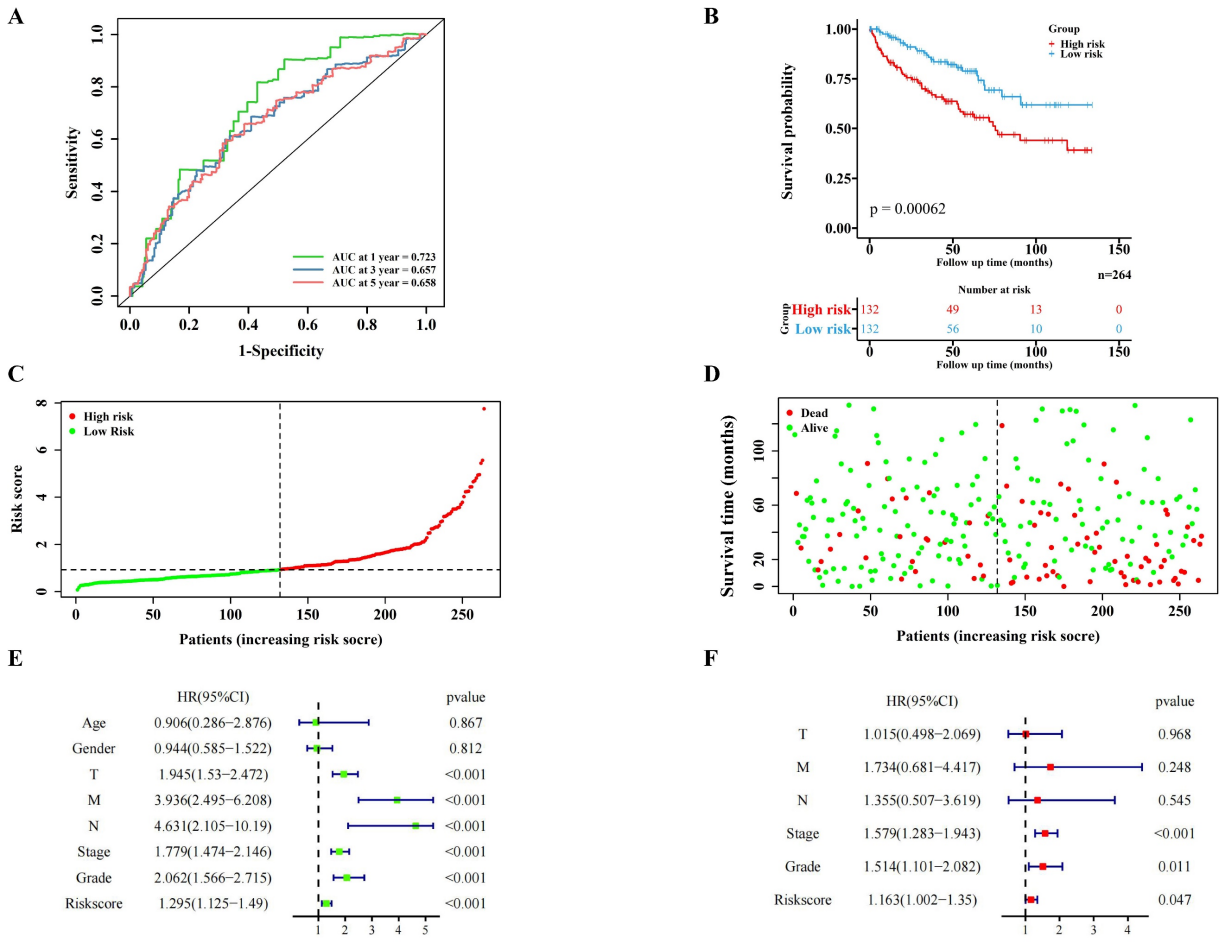
Validation of the PRGs prediction model in internal validation cohort. Time-dependent ROC curve (1 year-, 3year-, 5year-) of the PRGs prediction model in internal validation cohort. (B) The Kaplan-Meier survival analysis of the internal validation cohort patients in high-risk and low-risk groups. (C,D) Scatter diagram visualizing the distribution of the risk score and survival time of internal validation cohort patients in the prediction model. (E,F) Independent clinical indicators verified by the univariate and multivariate cox regression in our internal validation cohort.

The internal validation cohort of 264 cases of TCGA ccRCC data was used to verify the pyroptosis molecular biomarkers risk prediction model. The results demonstrated that the model was verified accurately in the internal training cohort, which is consistent with the training cohort (Figure 4A-F). Additionally, the immune-related scores of the internal validation cohort were also validated (Figure S4A-B; Figure S5B).

### ccRCC tissue array of EHSH was used for external validation

**Figure 5:**
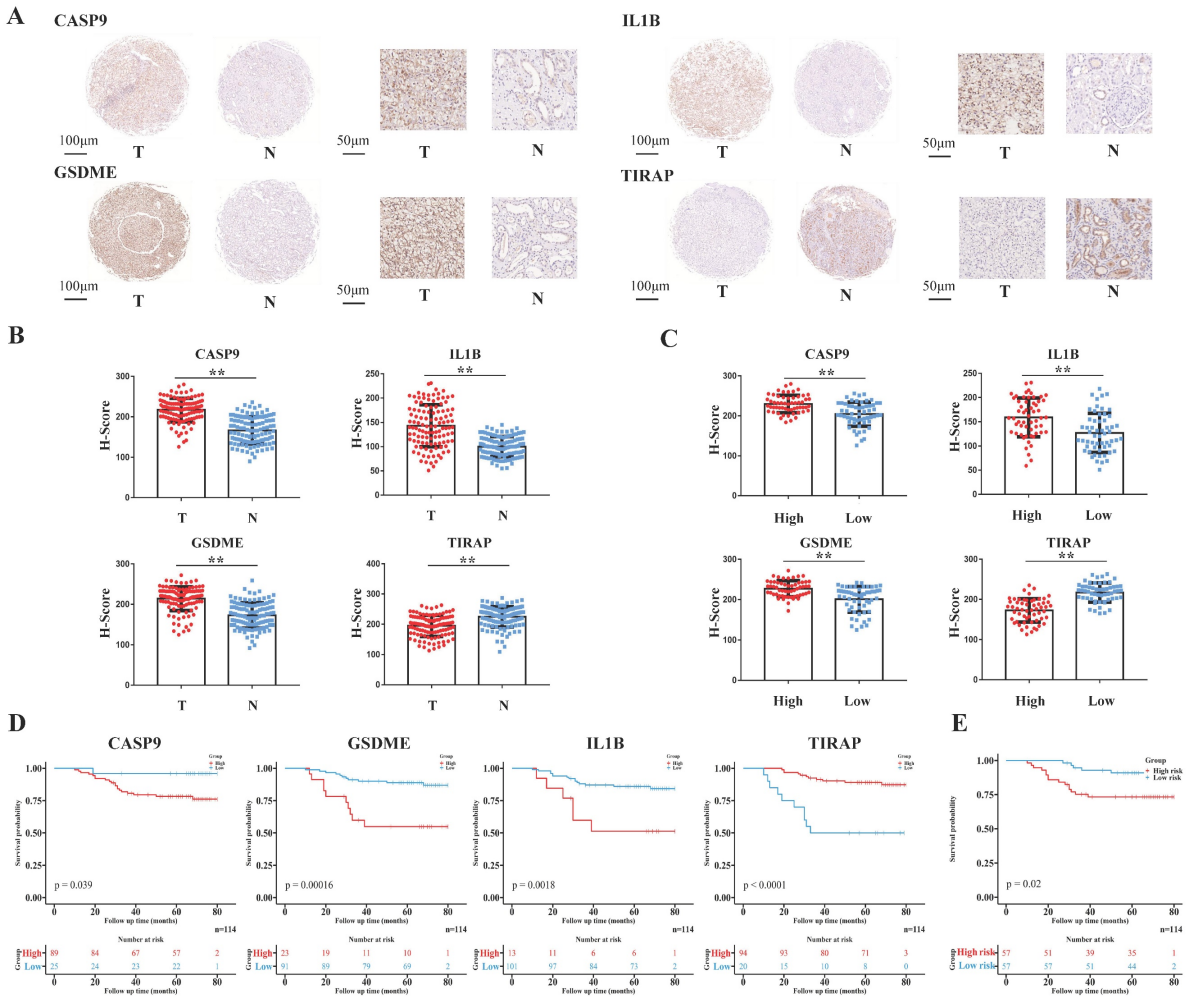
Validation of the PRGs prediction model in external validation cohort (EHSH cohort). (A) Immunohistochemistry displaying the protein expression of 4 PRGs in cancer and adjacent normal tissues in external validation cohort. (B) Bar plots showing the immunohistochemical scores of the 4 PRGs in cancer and adjacent normal tissues in external validation cohort, **P < 0.01. (C) Bar plots showing the immunohistochemical scores of the 4 PRGs in high-risk group and low-risk group patients in external validation cohort, **P < 0.01. (D) The Kaplan-Meier survival analysis of the 4 PRGs in the external validation cohort. (E) The Kaplan-Meier survival analysis of the two different groups (high-risk group and low-risk group) patients in the external validation cohort.

The tissue array of the 114 ccRCC patients from EHSH was used as our external validation of the model, and the four pyroptosis-related molecules (CASP9, GSDME, IL1B and TIRAP) of the model was IHC stained (Figure 5A). The risk score of EHSH patients were calculated according to the same formula of the training cohort and the EHSH patients were divided into two different groups (a high-risk score group and a low-risk score group). The results showed that the expression levels of CASP9, GSDME and IL1B in RCC were higher than those in the adjacent tissues, but the expression level of TIRAP in RCC was lower than that in the adjacent tissues (Figure 5B). Moreover, the IHC score of CASP9, GSDME and IL1B in high-risk score group patients were higher than those in low-risk score group patients, but the IHC score of TIRAP in high-risk score group patients were lower than that in the low-risk score group patients (Figure 5C). KM survival curve showed that the survival prognosis of patients with high expressions of CASP9, GSDME and IL1B was worse than that of patients with low expression, and the survival prognosis of patients with high expression of TIRAP was better than that of patients with low expression (Figure 5D). Meanwhile, the survival prognosis of the two risk score groups patients in EHSH cohort was also shown by KM curves (Figure 5E). These results demonstrated that the model was sensitive to verify the EHSH external validation cohort.

### The cellular function of PRGs in the model in ccRCC

**Figure 6:**
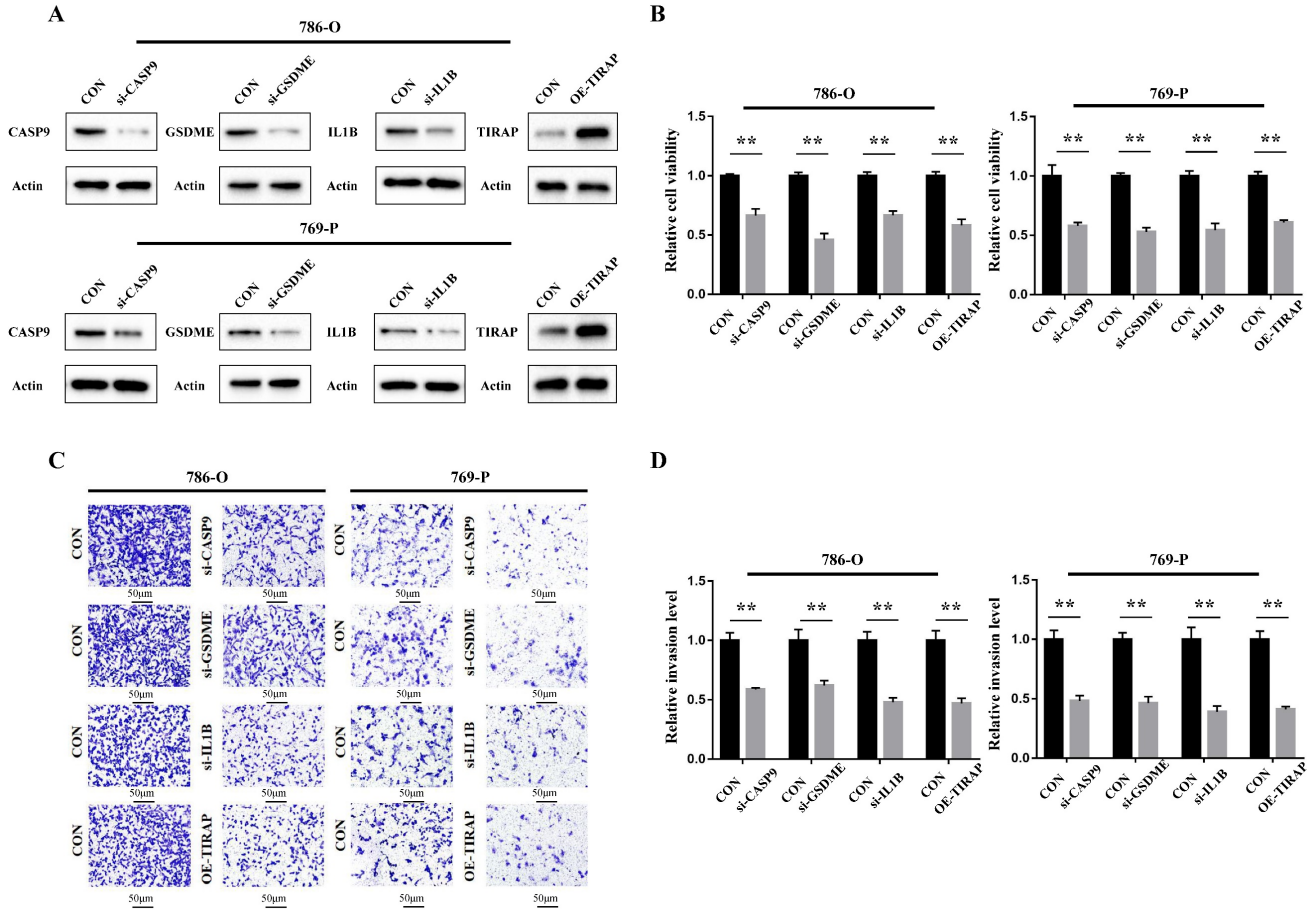
Oncogenic function of PRGs in the model in ccRCC. (A) Construction of ccRCC cell lines that interfere with 4 PRGs. (B) The ability of the proliferation of 786-O and 769-P cells after 4 PRGs knockdown or overexpression, **P < 0.01. (C,D) Detection of the invasion of 786-O and 769-P cells after 4 PRGs knockdown or overexpression by transwell assay, **P < 0.01.

The cellular function of the genes in the PRG prediction model was verified in ccRCC 786-O and 769-P cells. Firstly, siRNA of CASP9, GSDME and IL1B was constructed, and the overexpression plasmid of TIRAP was constructed and transfected into 786-O and 769-P cells. The constructed knockdown and overexpression cell line was verified by Western blot assay (Figure 6A). The results of the CCK8 cell proliferation experiment showed that the proliferation of ccRCC was decreased after silencing CASP9, GSDME and IL1B, and decreased after TIRAP overexpression (Figure 6B). The Transwell cell invasion experiment also showed that the ability of invasion in ccRCC cells was decreased after silencing CASP9, GSDME and IL1B, and decreased after TIRAP overexpression (Figure 6C-D). Hence, the four PRGs (CASP9, GSDME, IL1B and TIRAP) play as the oncogene or antioncogene in ccRCC, which affect the ability of the proliferation and invasion in ccRCC.

## Discussion

Surgery remains the mainstay of treatment for ccRCC. However, about 40% of patients with advanced ccRCC who underwent surgery eventually developed distant metastases (23,24, whose OS is usually poor 25. Some previous studies have demonstrated that even patients with the same TNM stage or risk factors had different clinical outcomes because of the molecular heterogeneity 26. Hence, it is important to identify novel prognostic molecular signatures,which may provide a suitable window of opportunity for ccRCC patients.

Like apoptosis, ferroptosis and autophagy, pyroptosis is a form of cell death, but one of the characteristics of pyroptosis is inflammatory cell death 13. Compared with apoptosis, pyroptosis is a kind of necrotic and inflammatory programmed cell death induced by inflammation [13]. The process of pyroptosis depends on caspase-1 27. Under external stimulation, the precursor of caspase-1 binds to pattern recognition receptor NLRP1 / 3 through adaptor protein ASC to form a high molecular compound known as an inflammatory body. Pores are formed when cells undergo pyroptosis, which allows for the release of lactate dehydrogenase and inflammatory cytokines 28. Previous studies have shown that the molecules related to pyroptosis are closely related to the proliferation, migration and tumor immunity of several tumors 29-35. However, the relationship between pyroptosis and ccRCC has not been thoroughly described.

In this study, we analyzed the TCGA database of ccRCC and randomly divided it into two groups: a training cohort (n=265) and an internal validation cohort (n=264). From 40 previously reported genes related to pyroptosis, four genes (CASP9, GSDME, IL1B and TIRAP) identified to be closely related to the survival prognosis of ccRCC patients were used to establish a molecular model of pyroptosis related to the prognosis of ccRCC. CASP9 (caspase-9) is a member of the Caspase family. It is reported that caspase-1-induced apoptosis involves the Bid-caspase-9-caspase-3 axis, which may lead to GSDME-dependent secondary pyroptosis 36. As reported in the previous study 14, GSDME (gasdermin E) belongs to the gasdermin family and is closely related to pyroptosis. IL1B, a member of the interleukin family, is secreted and released in the process of pyroptosis 13. TIRAP (TIR domain containing adaptor protein) is essential for inflammasome activation and can regulate the expression of caspase-1-related protease caspase-11 37.

The internal validation cohort also proved that the model was effective and accurate. Additionally, we used EHSH tissue (n=114) microarrays as the external validation cohort to perform IHC staining of the four pyroptosis genes (CASP9, GSDME, IL1B and TIRAP) in the model and analyzed the clinical information of the patients (Table 2). The results showed that the external validation cohort matched the model, which increased the richness of the model research. In addition to bioinformatics analysis, we constructed the si-RNAs of the 3 pyroptosis-related genes (CASP9, GSDME, IL1B) and OE-plasmid of the TIRAP in the model and transfected ccRCC 786-O and 769-P cells to verify their cellular functions. The results of the cellular functions experiments showed that the four PRGs in this research model could significantly affect the proliferation and invasion ability of ccRCC cells. Specifically, GSDME, CASP9 and IL1B could act as the oncogenes in ccRCC and TIRAP may act as the anti-oncogene in ccRCC. Additionally, our prediction model could also describe the close relationship between pyroptosis and ccRCC in tumor immunity, and the pyroptosis model score was highly parallel to the immune score. In view of the previous finding that pyroptosis has a far-reaching impact on the immune microenvironment 38,39, we wonder whether pyroptosis is closely related to immunotherapeutic targets such as PD1 and CTLA4, which will be further explored in our ongoing study through bioinformatics analysis and molecular biology experiments.

## Conclusion

In conclusion, this study established a risk model of pyroptosis-related molecular signatures to predict the prognosis of ccRCC, which may provide some new ideas for the molecular target therapy of ccRCC.

## Supplementary Material

Supplementary figures.

Supplementary tables.

## Figures and Tables

**Table 1:** Pyroptosis related genes.

Gene name	Full name	Gene Cards ID	Category
AIM2	Absent In Melanoma 2	GC01M159062	Protein Coding
CASP1	Caspase 1	GC11M105025	Protein Coding
CASP3	Caspase 3	GC04M184627	Protein Coding
CASP4	Caspase 4	GC11M104942	Protein Coding
CASP5	Caspase 5	GC11M104995	Protein Coding
CASP6	Caspase 6	GC04M109688	Protein Coding
CASP8	Caspase 8	GC02P201233	Protein Coding
CASP9	Caspase 9	GC01M015491	Protein Coding
ELANE	Elastase, Neutrophil Expressed	GC19P001191	Protein Coding
GPX4	Glutathione Peroxidase 4	GC19P001103	Protein Coding
GSDMA	Gasdermin A	GC17P042241	Protein Coding
GSDMB	Gasdermin B	GC17M039904	Protein Coding
GSDMC	Gasdermin C	GC08M129705	Protein Coding
GSDMD	Gasdermin D	GC08P143553	Protein Coding
GSDME	Gasdermin E	GC07M024699	Protein Coding
IL18	Interleukin 18	GC11M112143	Protein Coding
IL1B	Interleukin 1 Beta	GC02M112829	Protein Coding
IL6	Interleukin 6	GC07P022725	Protein Coding
NLRC4	NLR Family CARD Domain Containing 4	GC02M032224	Protein Coding
NLRP1	NLR Family Pyrin Domain Containing 1	GC17M005499	Protein Coding
NLRP2	NLR Family Pyrin Domain Containing 2	GC19P054953	Protein Coding
NLRP3	NLR Family Pyrin Domain Containing 3	GC01P247415	Protein Coding
NLRP6	NLR Family Pyrin Domain Containing 6	GC11P000269	Protein Coding
NLRP7	NLR Family Pyrin Domain Containing 7	GC19M054923	Protein Coding
NOD1	Nucleotide Binding Oligomerization Domain Containing 1	GC07M030424	Protein Coding
NOD2	Nucleotide Binding Oligomerization Domain Containing 2	GC16P050693	Protein Coding
PJVK	Pejvakin	GC02P178450	Protein Coding
PLCG1	Phospholipase C Gamma 1	GC20P041136	Protein Coding
PRKACA	Protein Kinase CAMP-Activated Catalytic Subunit Alpha	GC19M014354	Protein Coding
PYCARD	PYD And CARD Domain Containing	GC16M031201	Protein Coding
SCAF11	SR-Related CTD Associated Factor 11	GC12M045919	Protein Coding
TIRAP	TIR Domain Containing Adaptor Protein	GC11P126284	Protein Coding
TNF	Tumor Necrosis Factor	GC06P073386	Protein Coding
MEFV	MEFV Innate Immuity Regulator, Pyrin	GC16M006017	Protein Coding
HMGB1	High Mobility Group Box 1	GC13M030456	Protein Coding
LDHA	Lactate Dehydrogenase A	GC11P018394	Protein Coding
LDHB	Lactate Dehydrogenase B	GC12M021635	Protein Coding
NINJ1	Ninjurin 1	GC09M093121	Protein Coding
GZMA	Granzyme A	GC05P055102	Protein Coding
GZMB	Granzyme B	GC14M024630	Protein Coding

**Table 2:** Univariate and multivariate analyses in the training cohort and validation cohorts.

Cohort	Characteristic	Univariate				Multivariate		
		HR	95% CI	P value		HR	95% CI	P value
Training cohort	Age	1.384	0.192-9.973	0.747		-	-	-
	Gender	1.133	0.749-1.716	0.554		-	-	-
	T	1.917	1.531-2.401	< 0.001		0.956	0.581-1.575	0.861
	M	4.891	3.194-7.49	< 0.001		1.609	0.719-3.600	0.247
	N	3.039	1.113-8.299	0.03		2.077	0.696-6.194	0.19
	Stage	1.957	1.63-2.351	< 0.001		1.651	1.338-2.037	< 0.001
	Grade	1.876	1.494-2.357	< 0.001		1.337	1.014-1.765	0.04
	Riskscore	1.421	1.281-1.575	< 0.001		1.205	1.052-1.380	0.007
								
Internal validation cohort	Age	0.906	0.286-2.876	0.867		-	-	-
	Gender	0.944	0.585-1.522	0.812		-	-	-
	T	1.945	1.530-2.472	< 0.001		1.015	0.498-2.069	0.968
	M	3.936	2.495-6.208	< 0.001		1.734	0.681-4.417	0.248
	N	4.631	2.105-10.19	< 0.001		1.355	0.507-3.619	0.545
	Stage	1.779	1.474-2.146	< 0.001		1.579	1.283-1.943	< 0.001
	Grade	2.062	1.566-2.715	< 0.001		1.514	1.101-2.082	0.011
	Riskscore	1.295	1.125-1.49	< 0.001		1.163	1.002-1.35	0.047
								
External validation (EHSH) cohort	Age	0.622	0.209-1.849	0.393		-	-	-
	Gender	1.747	0.724-4.217	0.125		-	-	-
	T	2.297	1.535-3.437	< 0.001		0.394	0.143-1.058	0.065
	M	6.023	2.327-15.59	< 0.001		0.583	0.073-4.648	0.61
	N	2.941	1.077-8.034	0.035		0.565	0.139-2.305	0.426
	Stage	2.115	1.501-2.981	< 0.001		3.578	1.036-12.365	0.044
	Grade	2.822	1.76-4.526	< 0.001		2.119	1.09-4.121	0.027
	Riskscore	1.012	1.005-1.018	< 0.001		1.009	1.003-1.015	0.012
