# Supplementary material for: Novel model of pyroptosis-related molecular signatures for prognosis prediction of clear cell renal cell carcinoma patients
Source: Int J Med Sci. 2024 Jan 1;21(3):496–507. doi: 10.7150/ijms.88301 (PMC10797671; doi:10.7150/ijms.88301)
Supplement: Supplementary file 1 — Supplementary figures. [file ijmsv21p0496s1.pdf]

Figure S1 Construction of the prediction model depends NMF and survival analysis of 4 PRGs in the training cohort.

(A,B) The NMF method selects the number of groups in the model.

Figure S2 The survival analysis of 4 PRGs in the training cohort.

(A) The Kaplan-Meier survival analysis of the 4 PRGs in the training cohort. (B) The heatmap showing the expression of the 4 final PRGs in high-risk and low-risk groups.

Figure S3 The functional analysis of the two risk groups in training cohort.

(A,B) GO enrichment analysis and KEGG analysis showing the biological process enriched and signaling pathways enriched by DEGs in the training validation cohort.

Figure S4 The functional analysis of the two risk groups in internal validation cohort.

(A,B) GO enrichment analysis and KEGG analysis showing the biological process enriched and signaling pathways enriched by DEGs in the internal validation cohort.

Figure S5 The immune-related analysis of the two risk groups in training cohort and internal validation cohort.

(A) ssGSEA visualizing the training cohort enriched in immune-related pathways. (B) ssGSEA visualizing the training cohort enriched in immune-related pathways.

Figure S1

A

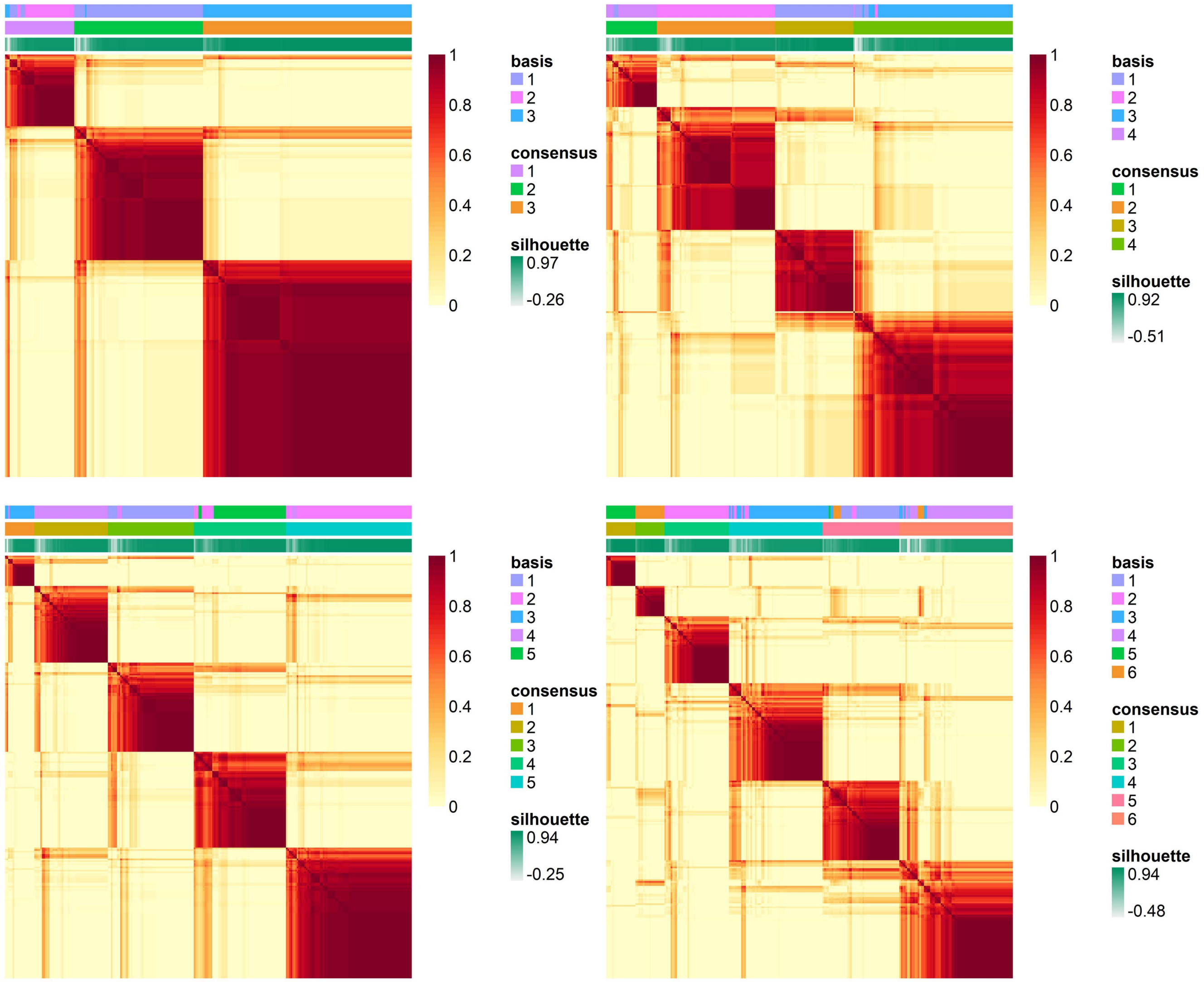

basis
consensus
silhouette
0.97
-0.26
0.92
-0.51
0.94
-0.25
0.94
-0.48

B

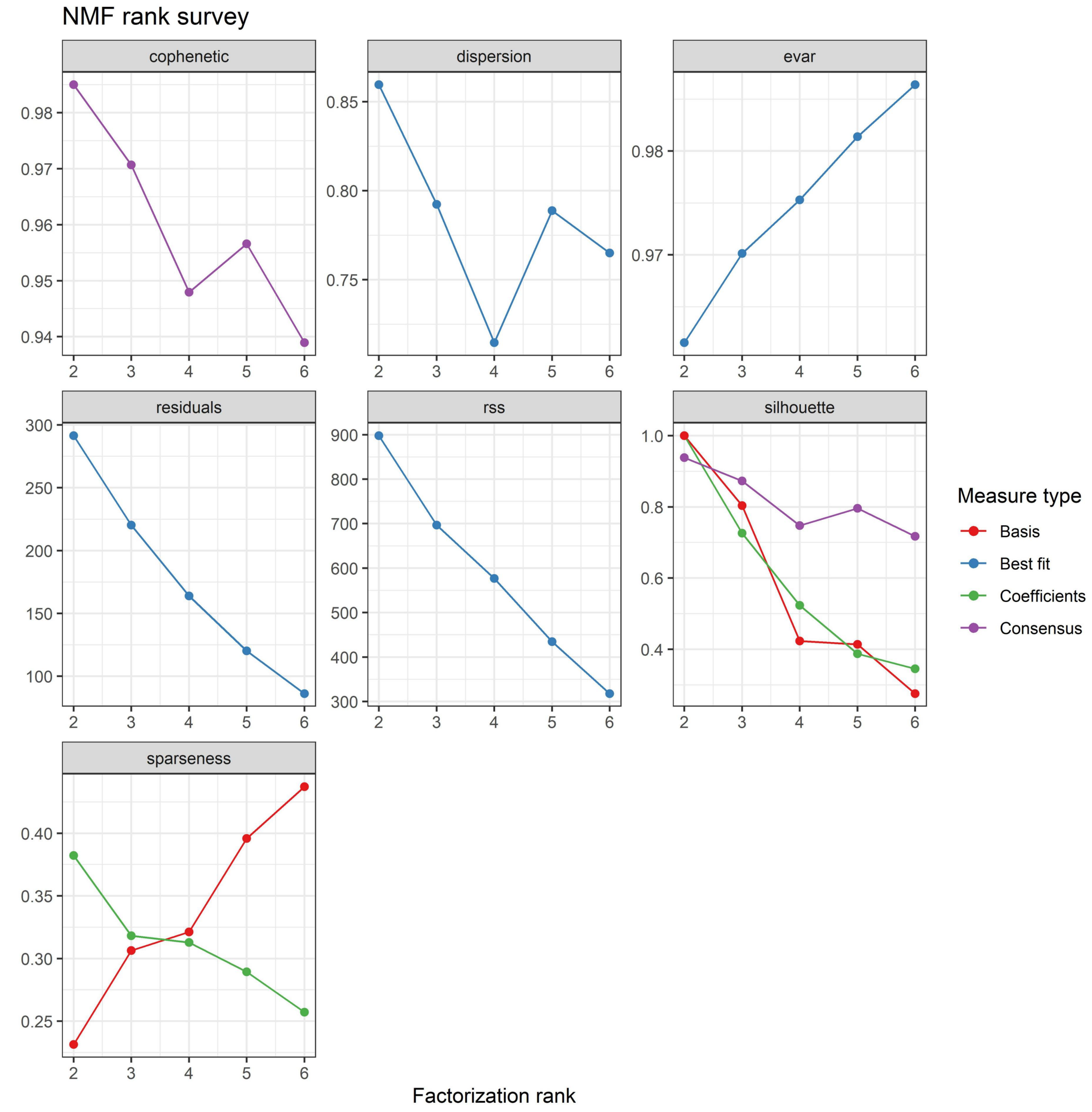

NMF rank survey
cophenetic
dispersion
evar
residuals
rss
silhouette
sparseness
Measure type
Basis
Best fit
Coefficients
Consensus
Factorization rank

Figure S2

A

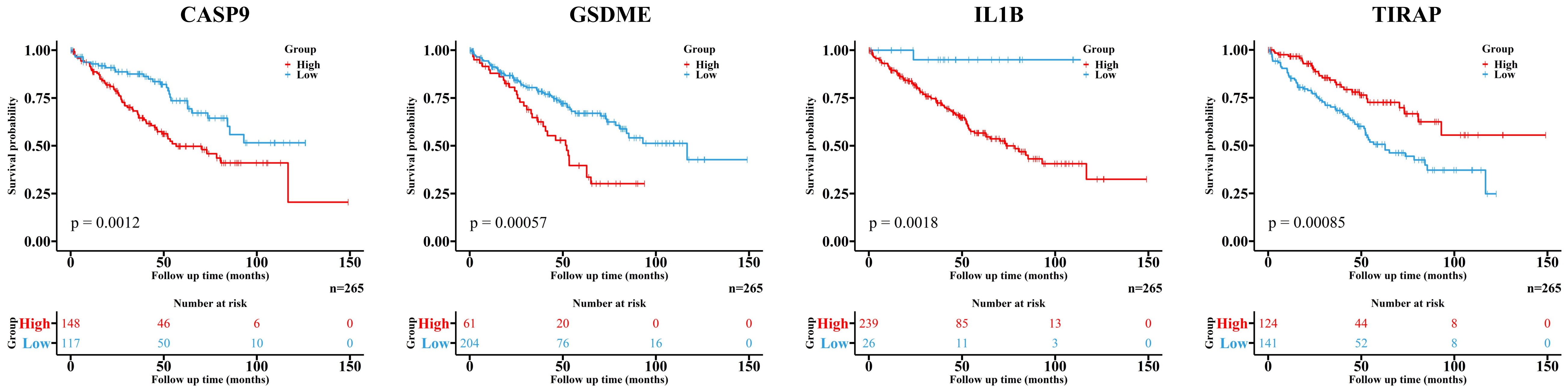
CASP9
GSDME
IL1B
TIRAP
Survival probability
Group
High
Low
p = 0.0012
p = 0.00057
p = 0.0018
p = 0.00085
Follow up time (months)
n=265
Number at risk
High 148 46 6 0
Low 117 50 10 0
High 61 20 0 0
Low 204 76 16 0
High 239 85 13 0
Low 26 11 3 0
High 124 44 8 0
Low 141 52 8 0

B

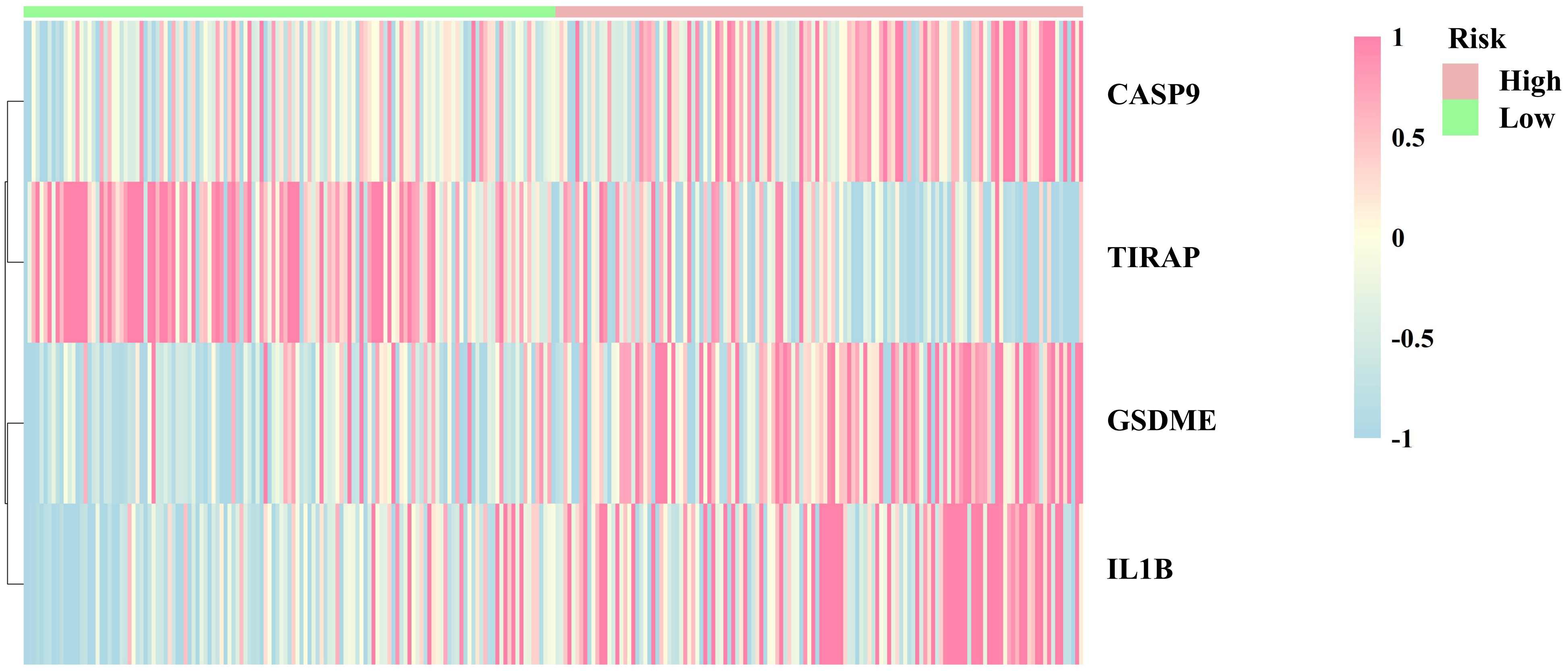
CASP9
TIRAP
GSDME
IL1B
1
0.5
0
-0.5
-1
Risk
High
Low

Figure S3

A

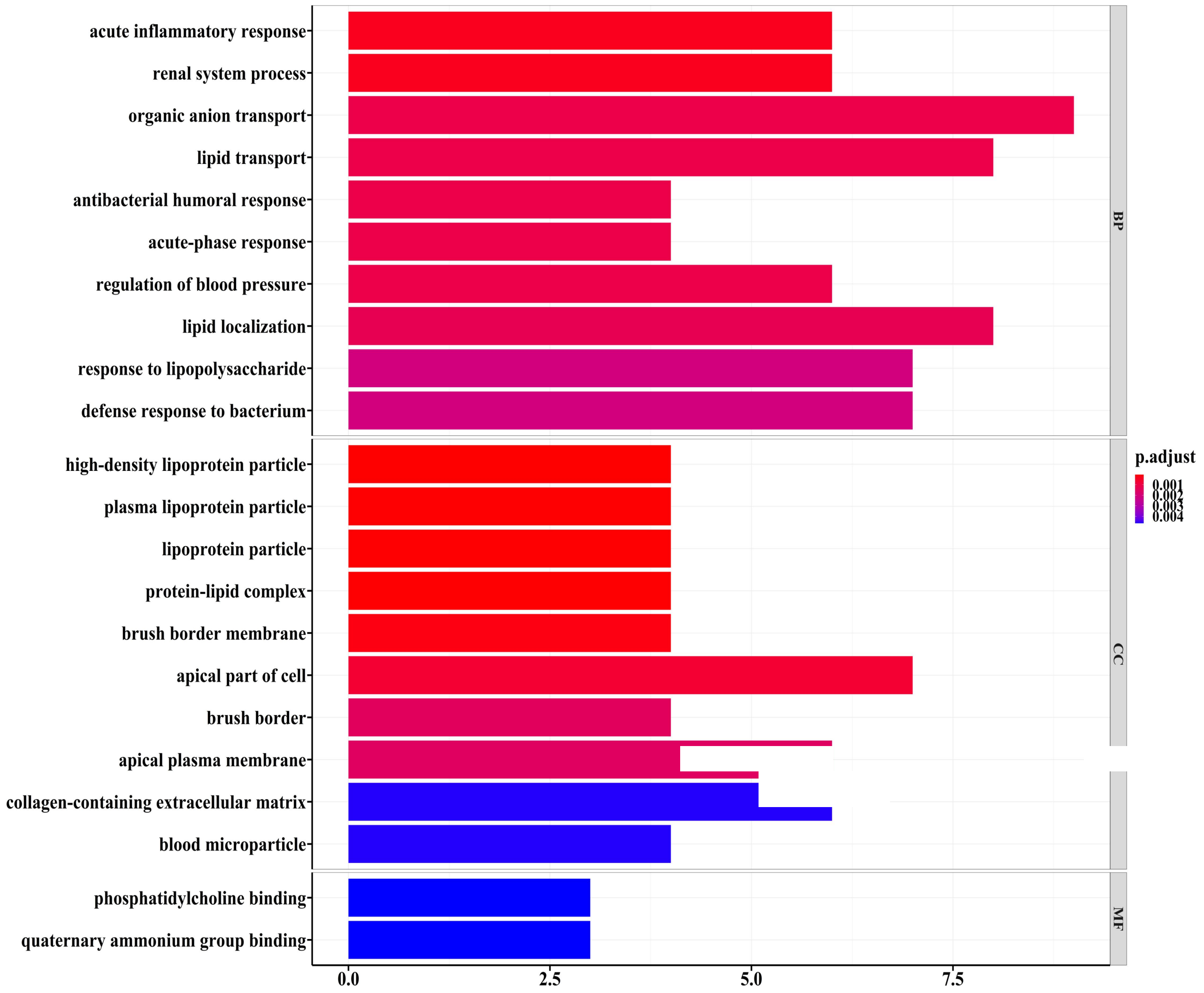
acute inflammatory response
renal system process
organic anion transport
lipid transport
antibacterial humoral response
acute-phase response
regulation of blood pressure
lipid localization
response to lipopolysaccharide
defense response to bacterium
high-density lipoprotein particle
plasma lipoprotein particle
lipoprotein particle
protein-lipid complex
brush border membrane
apical part of cell
brush border
apical plasma membrane
collagen-containing extracellular matrix
blood microparticle
phosphatidylcholine binding
quaternary ammonium group binding
BP
CC
MF
p.adjust
0.001
0.002
0.003
0.004
0.0
2.5
5.0
7.5

B

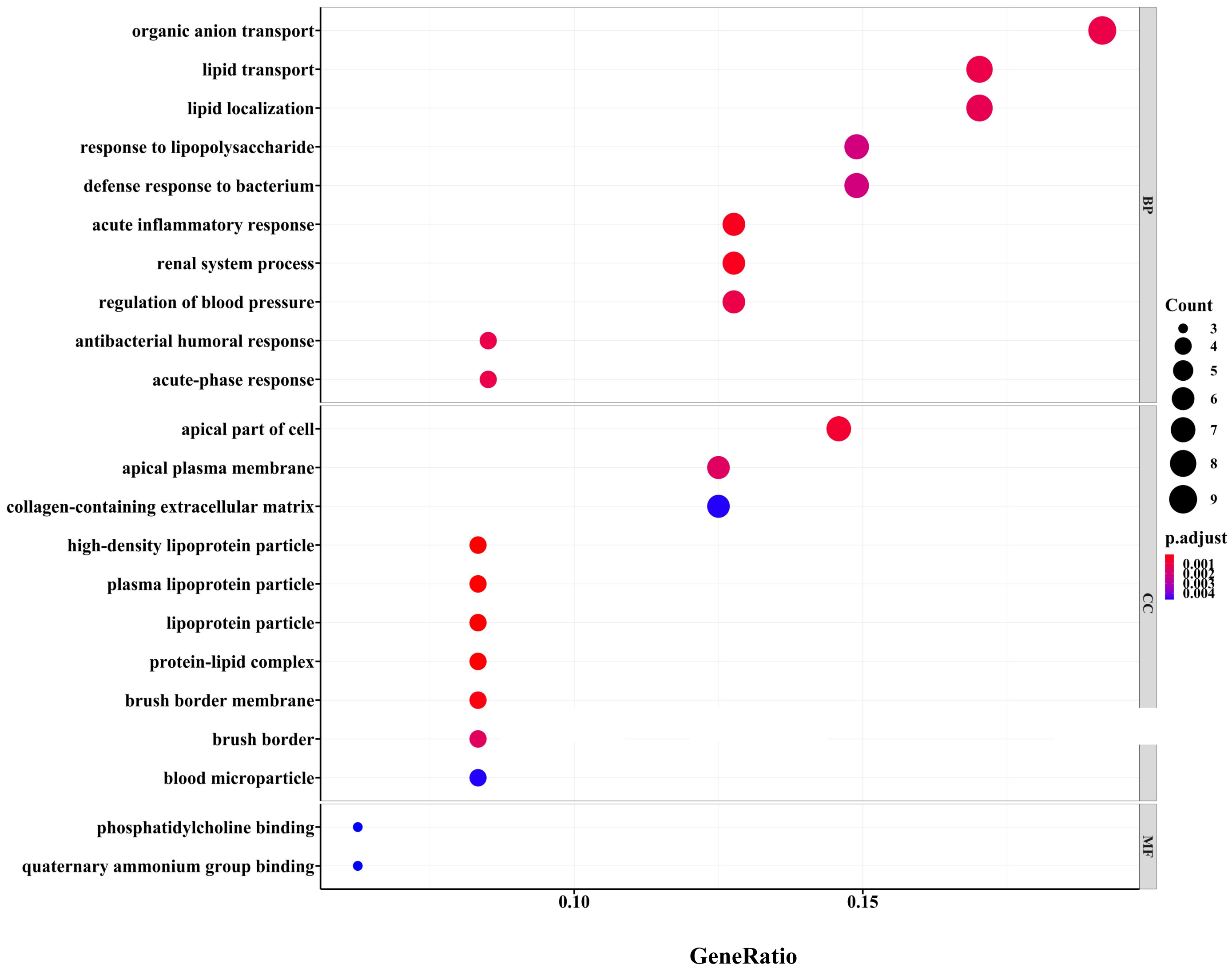
organic anion transport
lipid transport
lipid localization
response to lipopolysaccharide
defense response to bacterium
acute inflammatory response
renal system process
regulation of blood pressure
antibacterial humoral response
acute-phase response
apical part of cell
apical plasma membrane
collagen-containing extracellular matrix
high-density lipoprotein particle
plasma lipoprotein particle
lipoprotein particle
protein-lipid complex
brush border membrane
brush border
blood microparticle
phosphatidylcholine binding
quaternary ammonium group binding
BP
CC
MF
Count
3
4
5
6
7
8
9
p.adjust
0.001
0.002
0.003
0.004
0.10
0.15
GeneRatio

Figure S4

A B

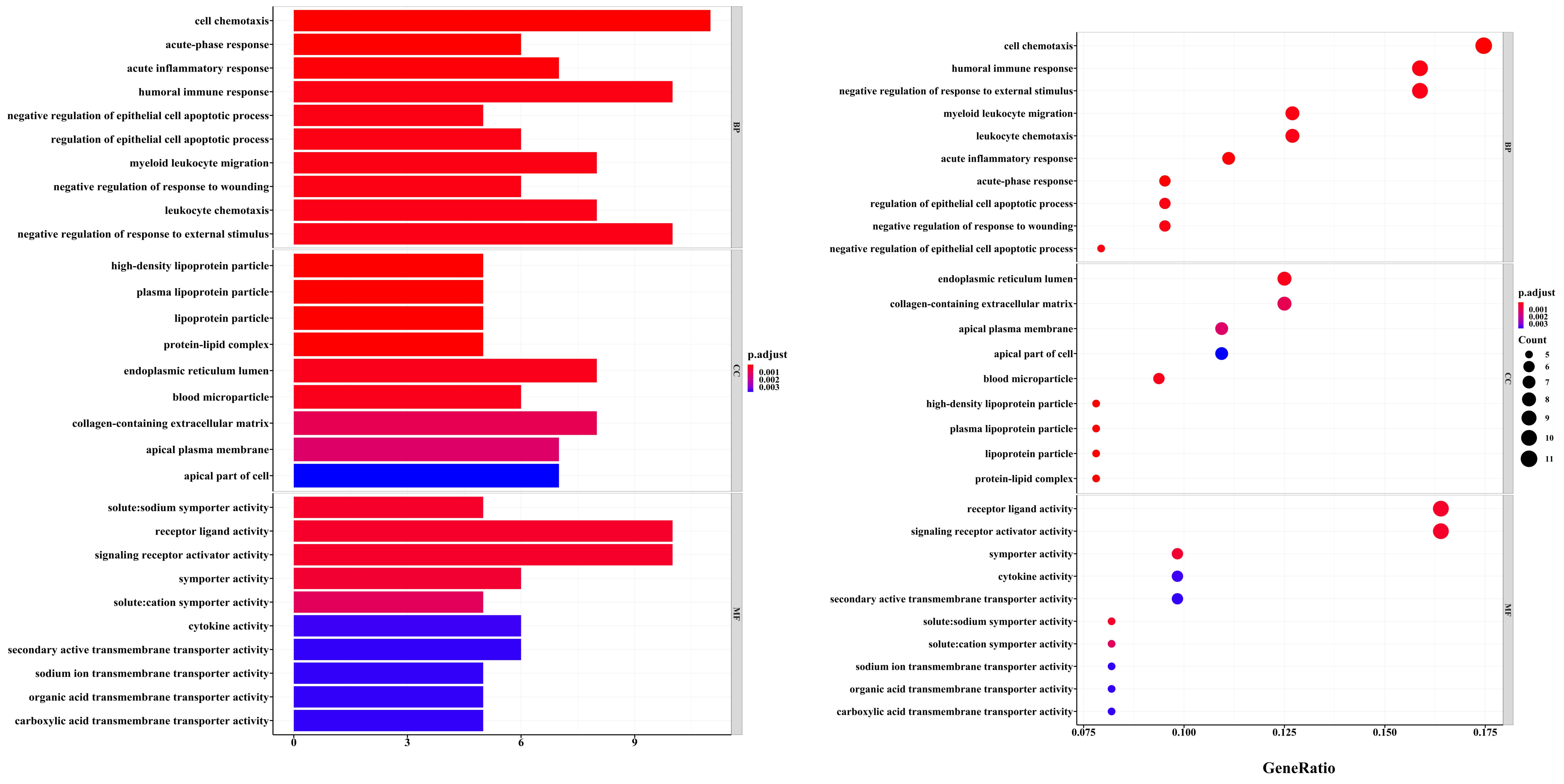

cell chemotaxis
acute-phase response
acute inflammatory response
humoral immune response
negative regulation of epithelial cell apoptotic process
regulation of epithelial cell apoptotic process
myeloid leukocyte migration
negative regulation of response to wounding
leukocyte chemotaxis
negative regulation of response to external stimulus
high-density lipoprotein particle
plasma lipoprotein particle
lipoprotein particle
protein-lipid complex
endoplasmic reticulum lumen
blood microparticle
collagen-containing extracellular matrix
apical plasma membrane
apical part of cell
solute:sodium symporter activity
receptor ligand activity
signaling receptor activator activity
symporter activity
solute:cation symporter activity
cytokine activity
secondary active transmembrane transporter activity
sodium ion transmembrane transporter activity
organic acid transmembrane transporter activity
carboxylic acid transmembrane transporter activity
BP
CC
MF
0
3
6
9
p.adjust
0.001
0.002
0.003
cell chemotaxis
humoral immune response
negative regulation of response to external stimulus
myeloid leukocyte migration
leukocyte chemotaxis
acute inflammatory response
acute-phase response
regulation of epithelial cell apoptotic process
negative regulation of response to wounding
negative regulation of epithelial cell apoptotic process
endoplasmic reticulum lumen
collagen-containing extracellular matrix
apical plasma membrane
apical part of cell
blood microparticle
high-density lipoprotein particle
plasma lipoprotein particle
lipoprotein particle
protein-lipid complex
receptor ligand activity
signaling receptor activator activity
symporter activity
cytokine activity
secondary active transmembrane transporter activity
solute:sodium symporter activity
solute:cation symporter activity
sodium ion transmembrane transporter activity
organic acid transmembrane transporter activity
carboxylic acid transmembrane transporter activity
BP
CC
MF
0.075
0.100
0.125
0.150
0.175
GeneRatio
p.adjust
0.001
0.002
0.003
Count
5
6
7
8
9
10
11

Figure S5

A

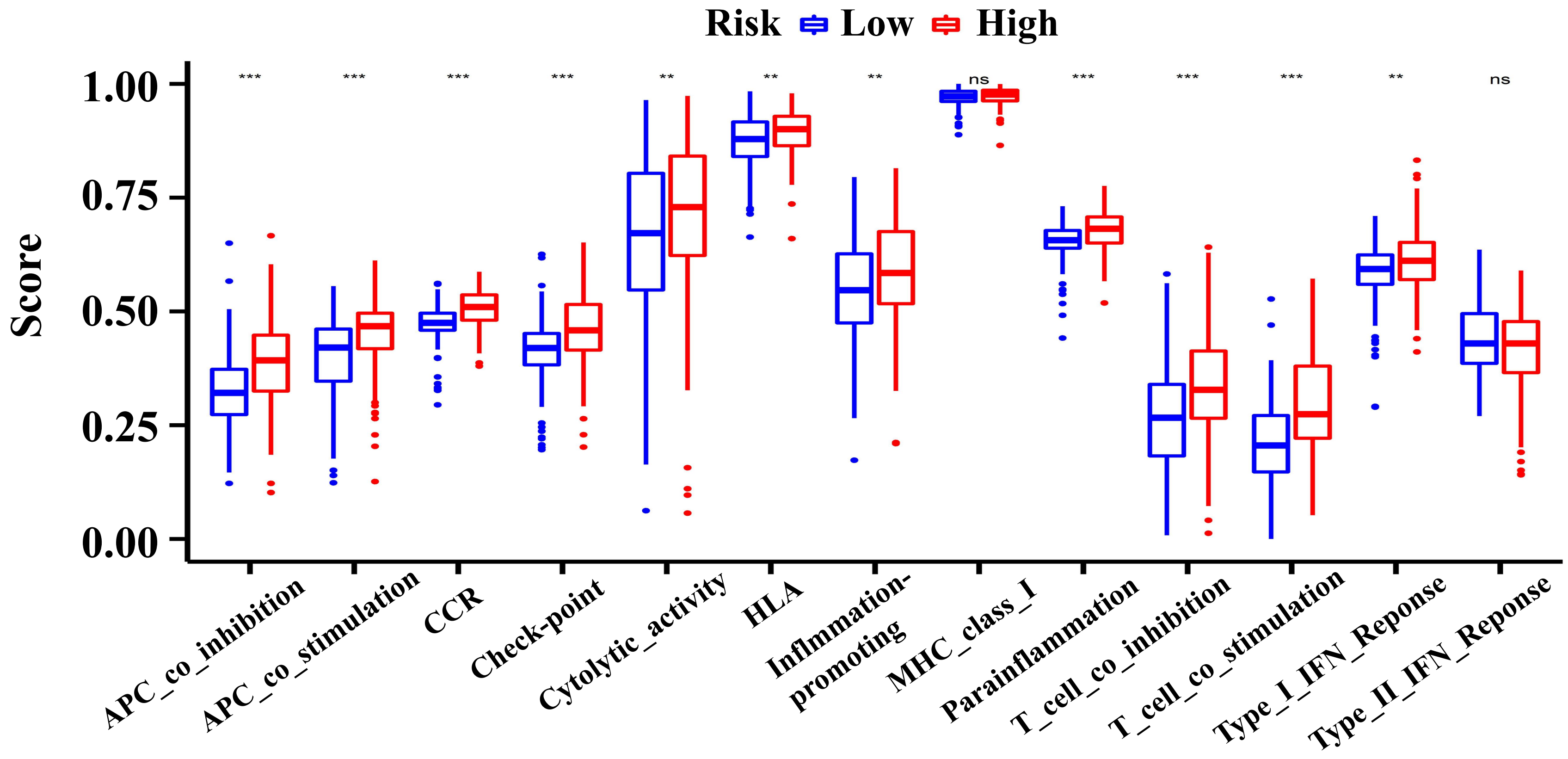
Risk Low High
Score
1.00
0.75
0.50
0.25
0.00
*** *** *** *** ** ** ** ns *** *** *** ** ns
APC_co_inhibition
APC_co_stimulation
CCR
Check-point
Cytolytic_activity
HLA
Inflammation-promoting
MHC_class_I
Parainflammation
T_cell_co_inhibition
T_cell_co_stimulation
Type_I_IFN_Reponse
Type_II_IFN_Reponse

B

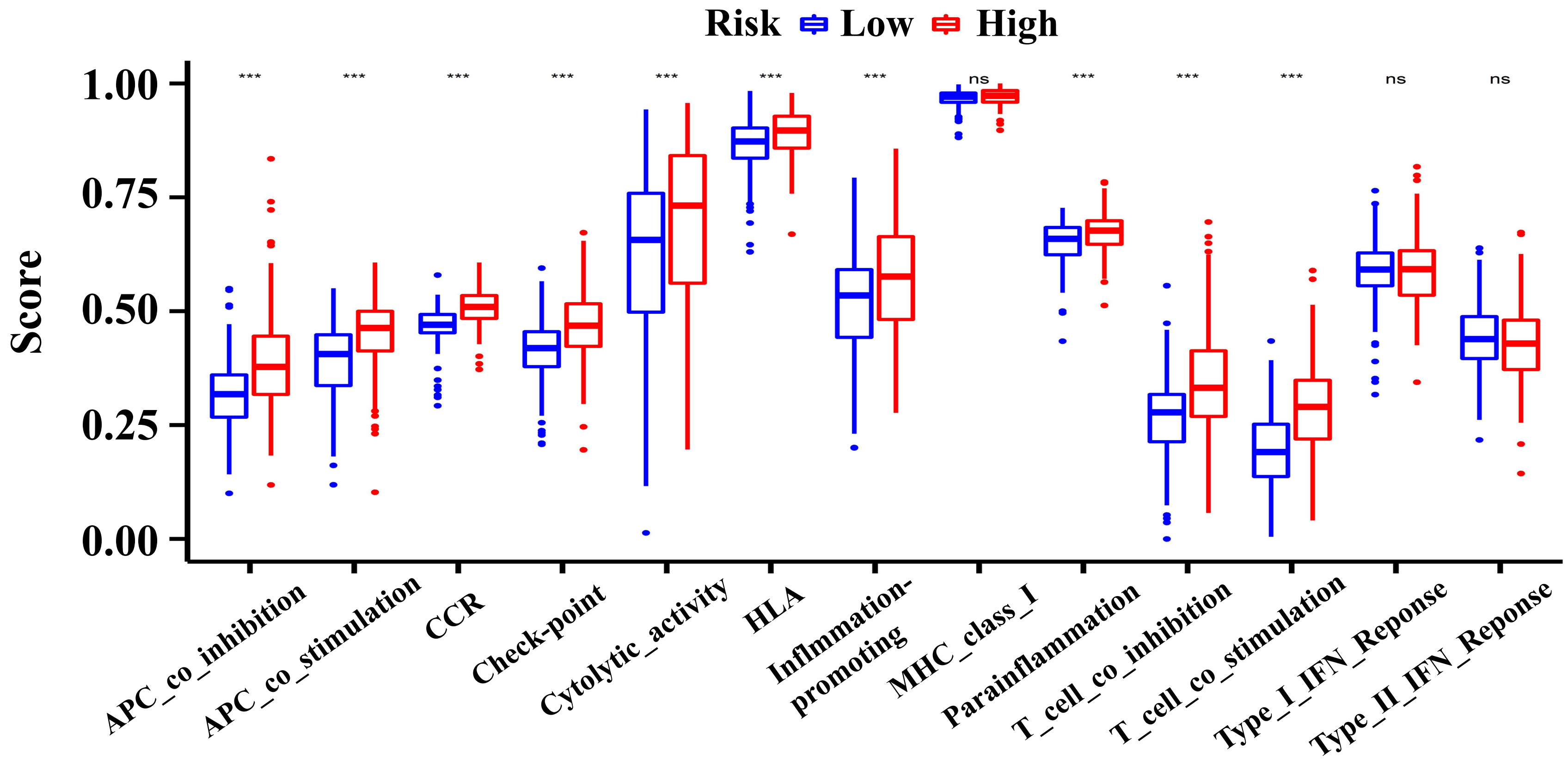
Risk Low High
Score
1.00
0.75
0.50
0.25
0.00
*** *** *** *** *** *** *** ns *** *** *** ns ns
APC_co_inhibition
APC_co_stimulation
CCR
Check-point
Cytolytic_activity
HLA
Inflammation-promoting
MHC_class_I
Parainflammation
T_cell_co_inhibition
T_cell_co_stimulation
Type_I_IFN_Reponse
Type_II_IFN_Reponse
